# Principles of practice for a whole school approach to self-harm: a qualitative study

**DOI:** 10.1186/s12889-025-25538-3

**Published:** 2025-12-17

**Authors:** Anne-Marie Burn, Hayley Gains, Poppy Hall, Joanna K. Anderson

**Affiliations:** Department of Psychiatry, University of Cambridge, Forvie Site, Robinson Way, Cambridge, CB2 0SZ UK

**Keywords:** Self-harm, Non-suicidal self-injury, NSSI, Schools, Young people, School staff, Principles of practice

## Abstract

**Background:**

Self-harm is common among adolescents and recognised as a significant public health issue. This study aimed to explore the perspectives of young people and school staff about how schools should respond to self-harm and subsequently develop principles of practice.

**Methods:**

Seven focus groups were carried out with school staff and young people. Qualitative data were thematically analysed using the Framework Method to generate themes [1, 2]. The findings were translated into actionable items by applying the Implementation in Schools Framework [3].

**Results:**

Staff and students had overlapping views about how schools should address self-harm. These were captured in three themes (1) Understanding the nature and scope of self-harm in schools, (2) Building whole school capacity to respond to self-harm, (3) Creating a supportive school environment. Principles of practice were generated to guide schools in their approach to self-harm.

**Conclusion:**

For many young people, schools are a key setting for early intervention in self-harm; however, staff often feel ill-equipped to respond and clear guidance is lacking. This study has developed evidence-informed principles of practice for UK schools, drawing on the perspectives of staff and young people to inform a whole school approach.

**Supplementary Information:**

The online version contains supplementary material available at 10.1186/s12889-025-25538-3.

## Introduction

Self-harm or non-suicidal self-injury (NSSI), is increasingly prevalent among young people and is widely recognised as a significant public health concern [4]. The term NSSI is often used in research to refer specifically to self-injury without suicidal intent, whereas self-harm is a broader term encompassing behaviours with or without suicidal intent. In the UK, self-harm is defined as ‘intentional self-poisoning or self-injury, irrespective of the apparent purpose’ [5], and is associated with a range of adverse outcomes, including disrupted education and employment trajectories and an elevated risk of suicide [4, 6]. Recent national survey data highlighted that 24% of 17-year-olds in the UK had self-harmed in the past year [7]. In England, the lifetime prevalence of self-harm among 15-year-olds rose sharply from 22% in 2014 to 34% in 2021 [8]. Research suggests that the COVID-19 pandemic further exacerbated mental health difficulties among young people, contributing to increased rates and severity of self-harm [9]. These trends are often associated with delays in help-seeking and persistent unmet mental health needs, including limited access to appropriate support and services [10, 11]. Early identification and timely intervention are crucial to improving outcomes [12]; however, many young people conceal their self-harm due to fear of stigma, being misunderstood, judged or labelled as ‘attention-seeking’ [10, 13–15]. As a result, only a small proportion of adolescents who self-harm present to hospital services [16].

UK Government policy highlights the role of schools in supporting young people’s mental health, including early intervention and prevention of self-harm and suicide [17]. Teachers and other education professionals are often among the first to notice signs of self-harm and are well-positioned to facilitate early intervention and referral to appropriate services [18]. Trusted relationships with school staff can play a vital role in encouraging help-seeking in adolescents. Research shows that young people are more inclined to seek help from a trusted staff member who listens, demonstrates empathy, and creates a supportive and safe environment [19, 20]. Additionally, staff have described how schools are increasingly acting as a “one stop shop” for parents seeking advice about their child’s mental health, often before approaching health professionals [21].

However, despite a general willingness to help, many school staff report feeling ill-equipped to respond to a student who is self-harming, citing a gap in training, insufficient knowledge and a lack of confidence in managing self-harm within the school setting [22, 23]. A recent survey found that only half of school staff received any training on self-harm, with just a fifth (22%) rating the training as highly adequate [24]. Limited staff knowledge can result in negative emotional reactions from staff when faced with a student’s self-harm, including panic, fear, or even repulsion [25]. Such responses may inadvertently discourage young people from seeking further help [10]. School staff have expressed a clear need for training and practical support tools to help them respond to and manage self-harm [19, 21]. Training programmes, particularly for pastoral staff, have shown promise in fostering more reflective and supportive school environments, by helping staff address their own concerns and challenge unhelpful attitudes towards self-harm [26].

Despite school staff expressing a clear need for improved knowledge and training on adolescent self-harm [24, 27], there remains a lack of effective, feasible and acceptable training programmes to support school staff [22, 28, 29]. A recent online training programme has been developed to address this gap [21, 30]. Moreover, although students consistently express a desire for open dialogue around self-harm, the topic remains largely absent from school curricula, and many schools still lack a dedicated policy on how to address self-harm [19, 21, 31]. A recent review highlighted the limited availability of school policies on self-harm and identified the need for evidence-informed guidelines to support schools in responding appropriately to instances of student self-harm [32].

In the UK, there is limited research and guidance for addressing self-harm in schools. A recent update of NICE guidance provides key advice on the practical steps educational professionals should take e.g. seek advice from a health professional, have a designated lead, make the young person aware of sources of support [5]. In New Zealand, Meinhardt et al. (2022) developed guidelines to support consistent and effective school-based responses to self-harm. Using a Delphi approach, drawing on input from mental health and education experts, the study produced a set of endorsed statements across key domains including staff training, responding to disclosures, post-incident support, and whole school responsibilities [33]. More recently, another New Zealand study identified key barriers and facilitators to implementing best-practice guidance for effective school responses to self-harm [34]. However, UK-based studies are currently lacking, and there is no equivalent evidence-informed framework to guide schools. Such a framework should be underpinned by a whole school approach that emphasises collaboration among staff, students, families and the wider community. This approach views the whole school as the unit of change and involves action across the curriculum, school ethos, and family and community partnerships [35].

The current study aimed to (i) explore the perspectives of young people and school staff about how schools can address self-harm and (ii) use the findings to develop a set of UK-specific principles of practice for supporting students who self-harm.

## Methods

### Design

This study was part of a broader programme of work that developed an online self-harm awareness training programme for secondary schools [19, 21, 29, 30]. We analysed qualitative data collected through focus groups with young people and school staff. Reporting has followed the Consolidated Criteria for Reporting Qualitative Studies (COREQ) guidelines (supplemental file 1).

### Ethical approval 

The authors assert that all procedures contributing to this work comply with the ethical standards of the relevant national and institutional committees on human experimentation and with the Helsinki Declaration of 1975, as revised in 2013. Ethical approval was granted by the University of Cambridge Department of Psychology Ethics Committee (refs: PRE.2022.05; PRE.2024.006).

### Participants and sampling

We conducted seven focus groups with 38 participants: five with secondary school staff (*n* = 27) and two with young people (*n* = 11). Staff were recruited from four state-maintained schools and represented a range of roles, including senior leadership (*n* = 5), teaching staff (*n* = 8), administrative and support staff (*n* = 3), and mental health and pastoral teams (*n* = 11). All schools had above-average rates of free school meal eligibility (>19%) and higher numbers of students with Special Educational Needs or Disabilities, both associated with poorer mental health. Three schools were mixed-sex and located in high-need areas of the East of England [36], while the fourth was a boys’ school in an inner-city London borough with above-average poverty levels.

Young people were recruited through one participating school and via social media. They were aged 14–21 years (median = 16) and had personal experience of self-harm and/or supporting a peer or sibling. Most (*n* = 9) were in secondary school, while one was attending university and another was in full-time employment.

### Procedure

We developed topic guides for each participant group in collaboration with public advisors, including four young people and a parent with lived experience, two school staff with mental health roles, and two representatives from a national charity specialising in self-harm awareness training in schools. Public advisors also reviewed and gave feedback on study documents and procedures to ensure that they were appropriate, acceptable and sensitive. Based on their input, we briefed participants prior to the focus groups about the nature of the discussion topics and clarified that they would not be asked to share personal experiences of self-harm.

At the beginning of the young people’s focus groups, we introduced short vignettes depicting scenarios of school staff responding to a student who was self-harming [37]. These served as a valuable tool to facilitate discussion of a sensitive topic, allowing participants to reflect without drawing directly on their own personal experiences. Topic guide questions for school staff explored their views on how schools should address self-harm, their own feelings (e.g. confidence and concerns about managing such situations), and their perspectives on staff training (see Supplementary file 2).

All participants were fully informed about the study and provided electronic consent before taking part; parental consent was obtained for those under 16 years of age. Focus groups were facilitated by AMB, an experienced qualitative researcher with expertise in working with young people who self-harm, supported by HG and PH. Two members of the team were clinically trained, and all members of the team were trained in safeguarding and risk management. Staff groups were conducted in schools during the school day. One young people’s group took place in school with a mental health lead present, while the other was conducted online to accommodate an evening time slot. At the start of the focus group, participants were reminded that they would not be asked to discuss their own self-harm experiences and that they could withdraw from the discussion at any point without giving a reason. It was explained that we would only disclose personal information if a participant shared something suggesting a risk of harm to themselves or others. Following each session, participants were debriefed both verbally and by email and provided with information about support resources.

Focus groups lasted about 1–2 hours, were audio recorded, and transcribed by a university-approved transcription service. Transcripts were checked, anonymised and stored securely in password-protected folders accessible only to the research team. Participants received a £20-per-hour online shopping voucher as a thank you for their time.

### Analysis

We analysed the qualitative data using the six stages of the Framework Method [1, 2], a pragmatic approach to analysis. Two researchers (AMB, HG) immersed themselves in the transcripts and independently coded a subset, working deductively with reference to the topic guide and inductively to capture meanings arising from the data. We compared and discussed our initial coding to develop a preliminary analytical framework, which we then applied to the remaining transcripts. We met weekly throughout the process, resolving any differences through discussion to reach consensus. This iterative approach enabled us to refine the framework constructively, incorporate new codes, and ensure consistency across the dataset, thereby strengthening the credibility and dependability of the process.

We created an Excel matrix, with rows representing focus groups and columns representing subthemes. Data were charted into this framework by AMB, HG and XS, with summaries supported by illustrative quotations to preserve the richness of participants’ accounts. In the final stage, we reviewed the data to identify cross-cutting patterns and generate higher-level themes.

To translate findings into practical recommendations, we mapped the themes onto the Education Endowment Foundation’s *Implementation in Schools Framework* [3]. Using a structured statement format (specifying what should be done, by whom, when, and how), we developed a set of evidence-informed principles of practice to enhance the likelihood of effective implementation in schools.

### Researcher background and reflexivity

Our study team all have psychology backgrounds, including two Chartered Psychologists and are mixed-methods researchers with expertise in young people’s mental health and school-based interventions. Two members of the team are senior researchers, and AMB has specialist expertise in qualitative and participatory methods. We met weekly throughout the analysis process to promote transparency and rigour, reflecting on how our disciplinary backgrounds and personal experiences might have influenced the interpretation of the data.

## Results

**Fig. 1:**
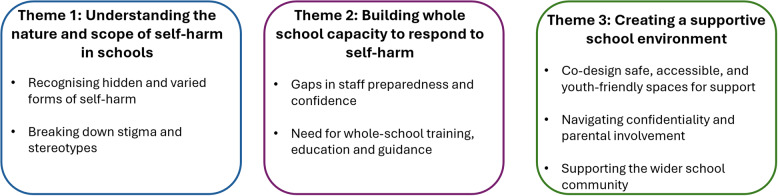
Main themes and subthemes

In this section, we present the main themes generated from the analysis, integrating the overlapping views and distinct perspectives of staff and young people: (1) Understanding the nature and scope of self-harm in schools, (2) Building whole school capacity to respond to self-harm, and (3) Creating a supportive school environment. While some subthemes reflected shared perspectives across both participant groups, some were primarily informed by staff data, particularly those focusing on organisational responses and staff preparedness. In contrast, subthemes related to help-seeking, trust and confidentiality were largely informed by young people’s accounts. The themes are summarised in the section below (see Figure 1), together with illustrative quotes from staff and young people. This section also presents the evidence-informed principles of practice developed from the themes.

### Theme 1: Understanding the nature and scope of self-harm in schools

#### Recognising hidden and varied forms of self-harm 

Both staff and young people viewed self-harm as a pressing and increasingly common issue in schools. Staff linked the rise of self-harm to several intersecting factors: high academic expectations, identity-related struggles, peer dynamics, the influence of social media and the lingering effects of the COVID-19 pandemic. Staff noted that self-harm is often “hidden”, particularly among high-achieving students, making it harder to identify those in need.

*Even though there’s no sign academically that that kid is struggling*,* they’re actually buckling under the worry and the weight of it all. (Staff)*

Mental health and pastoral staff emphasised that self-harm can take varied and often non-visible forms, highlighting the importance of recognising behaviours beyond cutting, such as ingestion of harmful substances, and of understanding the underlying emotional distress that drives these behaviours. There was broad consensus that improving staff awareness in this area is essential to ensure they are equipped to identify and support students who might otherwise remain unnoticed.

*“…digesting different types of toxic materials can be a form of self-harm. Recognising that you’re not always looking out for someone who’s got long sleeves. It’s different types of harm.” (Staff)*.

#### Breaking down stigma and stereotypes

Stigma was viewed as deeply rooted in generational and cultural attitudes, where mental health is not openly discussed and often perceived as “silly” or “shameful”. Staff expressed concern that some colleagues and parents hold judgemental attitudes about self-harm, and such beliefs could undermine relationships and deter young people from seeking help. They emphasised the need to challenge these attitudes, improve colleagues’ understanding, and adapt approaches to work sensitively with families from diverse backgrounds.

*…there’s generations where mental health wasn’t spoken about…And then it’s that element of they don’t know how to deal with it because their parents just shut it down. And so there needs to be more of a society training to deal with – not an epidemic*,* but there’s a lot more mental health issues now. (Staff)*

*I’ve been brought up in a culture that self-harm was this kind of an attention-seeking tactic…that culture very much still exists and I think it’s really important that we stamp that out. (Staff)*

Young people also noted that stigma, particularly the perception of self-harm as “attention-seeking” can discourage young people from seeking help. They felt schools should actively challenge these misconceptions by promoting education and open discussion to raising awareness.

*And the stigma behind it that some people might think that you’re only doing it for attention-seeking*,* and you don’t really want people to think that about you. (Young person)*

*I think the stigma with people our age is very much like they’re attention-seeking and that definitely is something that I think schools need to address more because the stigma around things really*,* really can make an impact on whether a student tells a teacher what’s going on or not. (Young person)*

Both young people and staff highlighted the need to challenge stereotypes. Staff working in a single-sex boys’ school pointed out that self-harm remains an issue among male students, despite the stereotype that it is predominantly a female problem. They noted that prevailing gender norms may contribute to the under-recognition of self-harm among boys and deter them from seeking support.

*…there’s some kind of gender divide*,* I mean*,* I think groups of boys in school*,* it’s not something that is spoken about (Young person)*.

*Because obviously this is a boys’ school*,* I don’t know if it’s more common in a girls’ school*,* like I think boys would try and hide it more. (Staff)*

### Theme 2: Building whole school capacity to respond to self-harm

#### Gaps in staff preparedness and confidence

Staff described the evolving role of teachers, noting that schools are increasingly functioning as “frontline services for students”. They reported being expected to manage complex mental health needs with limited guidance, fragmented support, and inconsistent training. Several recalled having to respond to self-harm incidents in the classroom without formal training or preparation, relying instead on instincts to remain calm and prevent escalation.

*I’ve had one girl who just did it with anything. I had to literally just go over and taking the object out of her hand and saying*,* “Right*,* we need to focus on our work now. But it was literally shocking just to think what she did. I’ve had no training. (Staff)*

Confidence in managing a self-harm disclosure varied widely across staff. Some described self-harm as a “scary” subject, primarily because of its close association with suicide. They reported feelings of guilt or denial after discovering a young person’s self-harm, as well as anxiety about “saying the wrong thing” and potentially triggering further harm.

*I thought it was a lot of pressure. You don’t want to do something wrong*,* you don’t know the wrong and right thing to do. Like you don’t want to make a situation worse*,* but then you don’t want to not help. So it’s like*,* for me*,* I just didn’t know the right or wrong way to go about things. (Staff)*

Teachers who felt unprepared described how limited training exacerbated anxiety and undermined their confidence in responding appropriately.

*I think mental health in general, school staff find it difficult if they've had no training or not thought about it,* *or if they think that speaking about it is likely to make it worse and therefore, don't ask the kind of direct questions you need to be able to make a risk assessment. (Staff)*

…*it is very shocking when a kid discloses it to you... I would like to know how to react to that information so that I don't do any more damage... I would like to be able to communicate better to pupils about these issues. (Staff)*

Staff confidence was often linked to previous mental health training. Those with relevant experience felt more able to engage in conversations with students, while others reported feeling unprepared and uncomfortable, sometimes avoiding the topic altogether. This lack of confidence was closely associated with limited knowledge, insufficient training and the absence of clear practical guidance.

*Because you might just freeze and your mind just goes blank and you don’t know what to actually say. You don’t want to say the wrong thing. So that would be good, just to have some guideline of what to say and questions. (Staff)*

Safeguarding provision was widely described as generic, with self-harm addressed only superficially and without practical strategies, leaving staff feeling under-prepared. A common concern was the absence of specific dedicated self-harm policies, with the issue typically subsumed under broader safeguarding procedures, as one staff member noted:

*Most schools don’t have a specific policy on self-harm...it kind of falls under the safeguarding procedures. (Staff)*

A lack of targeted guidance, combined with minimal training, was seen to undermine staff confidence and contribute to variable practice.

#### Need for whole school training, education and guidance

Staff emphasised the importance of a whole school approach to responding to self-harm, with training accessible to all staff, not only teachers or mental health teams. Non-teaching staff such as catering assistants, caretakers, and librarians were often described as having informal, trusting relationships with students, and therefore being well placed to notice early signs of distress.

*I think for something like self-harm, it should be an all-staff thing… they’re going to go to the person they feel most comfortable talking to. (Staff)*

*I work in the school library…I would like to be able to spot the warning signs. (Staff)*

Staff advocated for guidance on *what to say* and *how to say it* when a student discloses self-harm, going beyond procedural reporting. A whole school approach was viewed as essential for fostering shared understanding, ensuring consistency in responses and promoting a culture where safeguarding is a collective responsibility.

*I would feel really good about other people having the same knowledge…and that we have a shared knowledge base at a minimum. (Staff)*

Staff also emphasised the need for practical, accessible provision that could be integrated into existing safeguarding or Continuing Professional Development (CPD) sessions. Preferred formats included short, flexible modules delivered online or holding small, face-to-face groups, which would encourage discussion, reflection and sharing of good practice.

*It was good to be able to talk to other people… you got ideas from them or lived experiences from them, or how they deal with it. (Staff)*

Young people felt strongly that self-harm should be included in the curriculum and discussed more openly within schools to raise awareness and reduce stigma, which they saw as a barrier to help-seeking. They preferred that it be addressed directly rather than incorporated under general mental health education.

*Mental health is spoken about in a very generalised way. It would be beneficial to speak more openly and specifically about self-harm. (Young person)*

Young people also stressed the importance of being informed about relevant school policies and the support available to them, with several noting that uncertainty about where to seek help could delay or prevent disclosure.

*I think a lot of it is people don't know where to get that kind of support. So I think it's really important for schools to really put it out there and let students know where they should go to get that help. (Young person)*

They felt that while assemblies are helpful for sharing information about support, small group discussions were preferable for sensitive topics, as these provide a more comfortable space for conversations.

*Assemblies can be awkward…especially for more sensitive topics… you’re just sitting there awkwardly uncomfortable with it, then you’re not going to take in that information you need. It needs to be a comfortable environment. (Young person)*

Both staff and students emphasised the importance of clear, step-by-step guidance for what happens after a disclosure, with young people particularly highlighting the need to be actively involved in decisions about next steps to ensure that support is responsive and collaborative.

### Theme 3: Creating a supportive school environment

#### Co-design safe, accessible, and youth-friendly spaces for support

Participants emphasised that support for students who self-harm should be readily accessible, flexible and embedded in the school day. Both young people and staff felt it was important to provide quiet, private spaces where students could go for help without fear of being overheard or judged, particularly in moments of acute distress. Providing students with choice over *how* and *where* support takes place was seen as a way to increase their sense of control and reduce anxiety.

‘*Well, where would you like to have a chat?’ It gives them a bit of control over probably what is quite a scary situation. (Staff)*

Staff highlighted the importance of creating informal, youth-friendly spaces in which students feel safe to talk, as a way to build trust, reduce stigma around self-harm, and encourage early intervention.

Young people echoed this, describing the need for spaces that feel confidential and safe. However, there was a clear tension between staff and young people’s perspectives on what constitutes a safe space. While staff viewed designated ‘support rooms’ or‘ wellbeing hubs’ as visible and accessible sources of support, some young people felt these spaces could be intimidating or stigmatising, as others might notice and make assumptions about their mental health. Flexible, student-informed approaches were therefore seen as essential for encouraging help-seeking while minimising the risk of judgement or labelling.

*It’s got to be in their terms, that they feel comfortable that they have somewhere to go to talk particularly about whatever is on their mind at the time. (Staff)*

*Sometimes having a designated room can be more daunting…people assume they’re going there for a specific reason. (Young person)*

Across all participants, ‘trust’ emerged as central to encouraging help-seeking. While young people recognised the role of designated mental health staff, they stressed that familiarity and comfort would determine who they approached. Informal, everyday interactions - such as a chat with a form tutor during form time or lunchtime – were preferred over more formal sessions after school which could draw unwanted attention. Small gestures, like asking “How are you today?” were seen as important for building rapport and making support seem more accessible.

…*even just asking if the student is OK, just to let them know that someone cares…even if you don't already have that trust, you can build on that. (Young person)*

#### Navigating confidentiality and parental involvement

Transparency about confidentiality was a priority for young people, who wanted clear information on when and how parents would be involved. Uncertainty about these processes was seen as a source of anxiety that could deter disclosures. Young people felt that parents could struggle to accept or believe their child is self-harming.

* I think some parents would be like, "Oh, yeah, that would never happen to my child. Oh, they'd never do that," and brush it away. (Young person).*

*...you don't know what the relationship between the student and their parents is like, and having that shared at home could have giant repercussions. It could impact in a negative or a positive way, I guess, but if it impacts in a negative way, then it can make the situation so much worse. (Young person).*

Staff described the most challenging situations as those where students disclosed self-harm but do not want their parents informed. Safeguarding policies meant that staff had to act when a student’s safety was at risk, but explaining this to a student can be difficult and could undermine trust.

*I think the trickiest area for me when I've had those conversations is when students don't want their parents to know…my concern when you're sat having that conversation with a child and they do this and then they go, "Are you going to tell my parents?" and you have to then navigate your way around explaining why that has to happen. That's the tough bit. (Staff)*

Both staff and young people highlighted the importance of engaging parents constructively and sensitively. However, young people were particularly concerned that some home environments may not be supportive or may be the source of the young person’s distress.

…*we're assuming that the student has a good home life where they'll be supported through it, but that might not be the case, and it might be because of things at home. (Young person)*

This highlighted contrasting perspectives in how staff and young people viewed confidentiality and disclosure. Staff were concerned with meeting safeguarding obligations, while young people valued autonomy and trust in deciding when and how to involve parents.

#### Supporting the wider school community

Participants highlighted the need to extend support beyond the young person who is self-harming. For example, peers may sometimes learn that a friend is self-harming and disclose this to staff. Both staff and students emphasised the importance of reassuring peers that they had acted appropriately in seeking help, while managing confidentiality carefully. Staff expressed a need for clear protocols for handling peer-reported self-harm, recognising that insensitive responses could damage trust and potentially make the situation worse. Young people suggested that providing peers with general information about the support their friend would receive could help reduce uncertainty and anxiety, and encourage them to approach staff with concerns in the future.

*I would want to know how I could help my friend in that situation, because I think I'd feel quite lost. (Young person)*

Participants highlighted the importance of engaging parents constructively, providing accessible information and guidance on how to respond. This was seen as crucial given that self-harm is often hidden from parents, and first reactions may be angry, dismissive or disbelieving. Staff described the range of emotional responses they encounter and highlighted the need for greater parental support.

*We deal with a lot of parents who are naturally angry about what their child's done; they're disappointed, upset, angry, all of those kind of emotions, because they don't want their child to be doing that to themselves. I think that there's a certain degree of education for parents in supporting those young people, around what it is (Staff)*

Young people also noted that some parents might struggle to accept their child is self-harming, highlighting the need for awareness-raising and clear guidance to help parents recognise warning signs and respond constructively.

*I think with parents and figures of authority, I think the worry is a bad reaction because if they react badly, then you can't do anything about it and you've just got to deal with whatever the result of that may be. (Young person)*

Participants further emphasised the need of schools to be mindful of home circumstances. Not all families are supportive, and breaking confidentiality could have unintended consequences. As one young person explained:

*Telling one person such a big thing… if they go and tell loads of other people/tell your parents, it’s like all this information at once… at first it’s good to only tell one person and they keep that private. (Young person)*

To address these challenges, general education on self-harm for parents was considered essential, particularly during the early years of secondary school. Suggested approaches included parent workshops, written guides, and opportunities to speak with designated staff, enabling parents to feel more confident in having open, supportive conversations with their children about mental health.

 Staff discussed the emotional toll of responding to self-harm disclosures, particularly for those staff members with personal or family experience of self-harm. Such experiences could increase the emotional impact but also shape professional practice. Senior leaders emphasised the importance of signposting staff to appropriate support and resources to help them manage these demands:

*There is that fear from me as a leader… this could be really tough for someone to take on board. (Staff)*

###  Translating qualitative findings into actionable principles of practice for schools 

 From our analysis, we developed a set of behaviour-focused principles of practice to support schools in creating a whole school approach to self-harm. These principles were aligned with the Education Endowment Foundation’s Implementation in Schools Framework, based on an evidence review (3, 38). The framework conceptualises implementation as an iterative process comprising four stages (explore, prepare, deliver, sustain). For effective implementation, schools are encouraged to adopt three key behaviours (engage, unite, reflect). *Engage* involves meaningful consultation to shape overall direction, *unite* focuses on building buy-in and reinforcing why the new initiative is important, and *reflect* supports ongoing monitoring, adaptation and improvements to implementation. (Table 1)

**Table 1:** Principles of practice for schools

Theme	What	Who	When	How
Understanding the nature and scope of self-harm in schools	Whole school training for **all** school staff	Senior leadership	Prepare/Deliver	Unite staff around a shared understanding of self-harm. Train all staff (teaching & non-teaching) to recognise varied self-harm behaviours, risk factors, and how to respond appropriately and where to signpost.
	Develop school-wide self-harm policy	Senior leadership with whole school community input	Explore/Prepare	Self-harm policy tailored to school. Step-by-step post-disclosure guidance. Co-produce policy with students, staff and parents to promote shared understanding and consistency of response. Ensure the policy reflects the values of the whole school community.
	Raise awareness with staff, students, and parents to address misconceptions and stigma (e.g., “attention-seeking”, gender bias)	Wellbeing staff* & Mental Health Lead	Deliver/Sustain	Assemblies for general information on where to find support. Form time, posters, peer-led initiatives, parent workshops to provide information, support and anti-stigma messaging.
	Integrate self-harm awareness in curriculum	Senior leadership, teachers & students	Deliver/Sustain	Curriculum materials co-produced with students to be presented in small group PSHE or equivalent sessions; avoid large assemblies for sensitive topics.
Building whole school capacity to respond to self-harm	Induction & CPD** training and briefings for all staff	Senior leadership Wellbeing team & Mental Health Lead	Sustain	Scenario-based training for all school staff; quick-reference guides; reflection group discussions; access to follow-up support and resources provided.
	Ensure students know support options & policies	All school staff	Deliver/Sustain	Posters in visible locations, raise awareness of designated contacts, and methods to speak to staff. Engage with students to understand their ongoing level of awareness.
Creating a supportive school environment	Visible, accessible self-harm support within daily school routines	All school staff	Deliver/Sustain	Co-design confidential, stigma-free spaces with students. Engage with students to understand any barriers to help-seeking.
	Guidance for staff on managing confidentiality	Senior leadership	Prepare/Deliver/Sustain	Provide clear protocols on the limits of confidentiality.
	Support for peers following disclosures	All staff	Deliver/Sustain	Selected staff provide informal check-ins with peers. Reassure the peer student that they acted appropriately. Maintain confidentiality for peers to protect trust. Discuss with students how they would like the school to support them, giving them a sense of choice and autonomy.
	Monitor staff wellbeing and confidence	Senior leadership	Prepare/Deliver/Sustain	Access to reflective supervision, wellbeing resources, emotional support to manage the personal impact of disclosures.
	Engage parents with the school’s self-harm strategy and encourage positive, trusting relationships between staff and parents	All staff, Wellbeing Team and Mental Health Lead	Deliver/Sustain	Offer parents clear, practical information & resources on recognising signs of self-harm and responding constructively, especially at key transitions; provide workshops for parents.

*e.g. Counsellors, Safeguarding Leads, Mental Health Support Teams (MHSTs)

**Continuing Professional Development

## Discussion

 The current study explored the views and experiences of young people and school staff on how schools can address self-harm. Framework analysis generated three themes which represent overlapping views of staff and young people, including the need to understand the nature and scope of self-harm in schools, building a whole school approach, and creating a supportive school environment. Findings highlighted several ways in which schools can support students and have directly informed the principles of practice for a whole school approach to self-harm.

Staff and students perceived self-harm as a pressing and increasingly common issue, consistent with recent evidence of rising prevalence in the UK (39). Staff attributed this to multiple, interacting factors, such as high academic demands, identity related struggles, peer dynamics, social media and the effects of the COVID-19 pandemic. This suggests that many staff in our study were aware of the potentially complex and varied risk factors that can contribute to self-harm. Staff who had completed prior mental health training noted that self-harm behaviours are diverse, often hidden, and not readily recognised, supporting evidence that limited awareness of this range of behaviours may contribute to self-harm being frequently over-looked (40).

 Our findings also suggest that disclosure of self-harm and responses from others are influenced by broader cultural and social factors, such as stigma, stereotypes, and generational or cultural attitudes. Consistent with a previous study, young people described facing stigma and being labelled as attention-seeking (15), while wider cultural perceptions and societal norms across religion, media, and ethnicity can exacerbate feelings of guilt, shame or acceptance (41). 

 Participants in our study consistently endorsed implementing a whole school approach to self-harm that offers readily accessible and flexible support to all students who need it. Both staff and young people valued the availability of safe, quiet spaces within schools to speak with staff and receive support. However, some young people were cautious about designated wellbeing spaces, as they could make young people feel exposed and stigmatised when support is too visible. Other studies have shown that young people often feel uncomfortable sharing private thoughts and feelings in the presence of peers, as this makes them feel more vulnerable (19, 42). Moreover, being seen speaking with designated pastoral staff can carry negative labels such as ‘troublemakers’ and ‘difficult’, creating concerns around stigma (43).

In our study, trust and existing relationships were seen as key. Findings highlighted that young people prefer to speak with a familiar staff member they trust, rather than a formal ‘point person’, reflecting broader concerns around confidentiality and privacy when speaking about mental health to a staff member they do not know well (44). School staff in other studies have emphasised the ongoing need to build and maintain trusting relationships so that students feel comfortable seeking support at school when problems arise (45). Our findings highlight the need to involve young people in decisions around parental involvement, ensuring a balance between safeguarding responsibilities and student preferences. Consistent with prior studies, staff supported a student-led approach in which students are consulted on the timing, content and extent of how much information is disclosed (46, 47).

 Participants described responding ‘on their feet’ to disclosures without training or clear guidance, with self-harm most often treated solely as a safeguarding issue. These accounts reflect wider evidence that school staff feel underprepared, lacking in knowledge and confidence to respond to self-harm, and call for targeted training and consistent school-level policies (22, 28, 29). Staff in our study advocated for a unified approach, shared practice, and training accessible to all members of the school community. This is consistent with evidence showing that staff members without a mental health support role are often the first to identify signs of self-harm (48), and that equipping all staff with basic skills to recognise mental health difficulties and provide appropriate support is essential (46). Recent work also highlights the importance of implementing best practice guidelines consistently across schools (34). Young people in our study emphasised the value of including self-harm within the curriculum to raise awareness, reduce stigma and encourage help-seeking, echoing calls for structured education on self-harm in schools (19). However, the design and delivery of education materials should be co-produced with students to ensure relevance and engagement. 

 Our study also draws attention to the need for schools to support the wider school community following incidents of self-harm. Staff sought clearer guidance on responding to peer-reported self-harm and for supporting students who confide about a friend’s behaviour. This aligns with an earlier study that young people want education on recognising signs of self-harm and how to respond to a disclosure from peers (19). Both staff and young people highlighted that peers who disclose another student’s self-harm also require support and reassurance that they have done the right thing. Parents were also identified as needing tailored information and guidance in how to recognise signs of self-harm and respond constructively to their child. This reflects previous research showing that parents value support related to communication, psychoeducation, emotional regulation and parenting strategies (49). Finally, staff in our study raised the personal and emotional impact of managing student self-harm, particularly for those with lived experience. Staff in leadership roles emphasised the need for resources to support staff wellbeing, echoing findings that staff often experience lasting emotional strain and find it difficult to ‘switch off’ outside of work. These insights highlight the importance of access to external support (48).

A participatory approach is critical in school-based mental health research to ensure that policies and guidance are grounded in the lived experiences and perspectives of students and staff. Such involvement helps ensure that recommendations are contextually relevant, feasible within school systems, and sensitive to the relational dynamics that influence help-seeking and access to support. Drawing on these perspectives, this study developed a set of evidence-informed principles of practice specific to self-harm, aligned with broader evidence on effective implementation in schools. These principles move beyond general recommendations for staff training or policy development by offering practical guidance on how schools can create supportive environments that foster early identification and compassionate responses. They are also intentionally adaptable, providing a flexible framework that can be applied across diverse educational contexts - an essential feature given the variation in school resources, student needs, and local service provision.

### Limitations 

A strength of this study is that participants were drawn from areas with high levels of mental health need, including schools with above average rates of free school meal eligibility in both urban and rural settings. However, recruitment was limited to the East of England and London and may not reflect the perspectives of staff and young people in other parts of the UK. While the findings are grounded in the perspectives of school staff and young people in England, they may be applicable to similar educational contexts with appropriate adaptation. Moreover, the themes of stigma, staff confidence and the importance of whole school approaches are consistent with international literature, suggesting the findings may have broader relevance beyond the UK context. We also acknowledge the imbalance in sample sizes between staff and young people, meaning themes may be more reflective of staff perspectives. However, the concept of *information power* (50) underpinned our analytic approach, and the adequacy of the sample was supported by the richness and relevance of the data in addressing the study aim. 

 Future research should focus on developing and evaluating interventions for parents that promote constructive responses to self-harm disclosures, as well as interventions to support staff wellbeing in managing the emotional demands of these situations. In addition, there is a need to design resources for siblings and peers of young people who self-harm as they may also be affected. Further work should explore young people’s views in greater depth to inform the development of student-centred spaces and policies for responding to self-harm within schools, ensuring that practices are acceptable and appropriate. Finally, research should examine how schools can engage parents and students in co-producing a whole school policy on self-harm, to address differences in perspectives and foster a shared understanding of how best to prevent and respond to self-harm.

## Conclusions

This study makes an important contribution by developing evidence-informed principles of practice for UK schools, grounded in the perspectives of staff and young people. While schools are a key setting for early intervention and prevention of self-harm, staff often feel underprepared due to limited training, knowledge and guidance. By highlighting these gaps and proposing whole school principles of practice, this study provides a foundation for improving staff confidence, fostering supportive environments and ensuring that school responses to self-harm are both effective and acceptable to young people.

## Supplementary Information

Supplementary Material 1

Supplementary Material 2

## Data Availability

The datasets generated and/or analysed during the current study are not publicly available due to risk of identifying participants but are available from the corresponding author on reasonable request.

## Abbreviations

CPD: Continuing Professional Development
MHSTs: Mental Health Support Teams
PSHE: Personal, Social, Health and Economic education

## References

[1] Ritchie J, Lewis J, McNaughton Nicholls C, Ormston R. Qualitative research practice: a guide for social science students and researchers. Sage Publications London; 2013.

[2] Gale NK, Heath G, Cameron E, Rashid S, Redwood S. Using the framework method for the analysis of qualitative data in multi-disciplinary health research. BMC Med Res Methodol. 2013;13(1):117. 24047204 10.1186/1471-2288-13-117 PMC3848812

[3] Sharples J, Eaton J, Boughelaf J. A School’s Guide to Implementation. Guidance Report; 2024.

[4] Hawton K, Saunders KEA, Connor RCO. Self-harm and suicide in adolescents. Lancet. 2012;379:2373–82. 22726518 10.1016/S0140-6736(12)60322-5

[5] NICE. Self-harm: assessment, management and preventing recurrence. 2022. 36595613

[6] Borschmann R, Becker D, Coffey C, Spry E, Moreno-Betancur M, Moran P, et al. 20-year outcomes in adolescents who self-harm: a population-based cohort study. Lancet Child Adolesc Health. 2017;1(3):195–202. 30169168 10.1016/S2352-4642(17)30007-X

[7] Patalay P. Psychological distress, self-harm and attempted suicide in UK 17-year olds: prevalence and sociodemographic inequalities. Br J Psychiatry. 2021;219(2):437–9. 33436118 10.1192/bjp.2020.258

[8] Hulbert SE, Ferris T, Hrytsenko E, Kendall V. S. HBSC England National report: findings from the 2001–2022 HBSC study for England. University of Kent; 2023.

[9] Trafford AM, Carr MJ, Ashcroft DM, Chew-Graham CA, Cockcroft E, Cybulski L et al. Temporal trends in eating disorder and self-harm incidence rates among adolescents and young adults in the UK in the 2 years since onset of the COVID-19 pandemic: a population-based study. Lancet Child Adolesc Health. 2023;0(0):544–54. 10.1016/S2352-4642(23)00126-8 37352883

[10] Rowe SL, French RS, Henderson C, Ougrin D, Slade M, Moran P. Help-seeking behaviour and adolescent self-harm: a systematic review. Aust N Z J Psychiatry. 2014;48(12):1083–95. 25335872 10.1177/0004867414555718

[11] Gunnell D, Appleby L, Arensman E, Hawton K, John A, Kapur N, et al. Suicide risk and prevention during the COVID-19 pandemic. Lancet Psychiatry. 2020;7(6):468–71. 32330430 10.1016/S2215-0366(20)30171-1 PMC7173821

[12] Nurkhodjaev SB, Abdullaeva S. Early detection and prevention of suicidal behavior in adolescents. Indian J Forensic Med Toxicol. 2020;14(4):7258–63.

[13] Nada-raja S, Morrison D, Skegg K. A population-based study of help-seeking for self-harm in young adults. Aust N Z J Psychiatry. 2003. 10.1046/j.1440-1614.2003.01252.x. 14511089 10.1046/j.1440-1614.2003.01252.x

[14] Curtis S, Thorn P, McRoberts A, Hetrick S, Rice S, Robinson J. Caring for young people who self-harm: a review of perspectives from families and young people. Int J Environ Res Public Health. 2018. 10.3390/ijerph15050950. 29747476 10.3390/ijerph15050950 PMC5981989

[15] Lloyd B, Blazely A, Phillips L. Stigma towards individuals who self harm: impact of gender and disclosure. J Public Ment Health. 2018;17(4):184–94.

[16] Hawton K, Rodham K, Evans E, Weatherall R. Deliberate self harm in adolescents: self report survey in schools in England. BMJ. 2002;325(7374):1207–11. 12446536 10.1136/bmj.325.7374.1207 PMC135492

[17] Department for Health, Education Df. Transforming children and young people’s mental health provision: a green paper Impact Assessment Summary. United Kingdom Government; 2017. pp. 1–32.

[18] Newlove-Delgado T, Moore D, Ukoumunne OC, Stein K, Ford T. Mental health related contact with education professionals in the British child and adolescent mental health survey 2004. The Journal of Mental Health Training, Education and Practice. 2015;10(3):159–69.

[19] Colville L, Anderson JK, Burn A-M. How schools can respond to pupils who self-harm: a qualitative study with young people and school staff. Emotional and Behavioural Difficulties. 2024;29(3–4):123–37.

[20] O’Reilly M, Svirydzenka N, Adams S, Dogra N. Review of mental health promotion interventions in schools. Soc Psychiatry Psychiatr Epidemiol. 2018;53(7):647–62. 29752493 10.1007/s00127-018-1530-1 PMC6003977

[21] Burn A-M, Hall P, Anderson JA, Web-Based. Training program for school staff to respond to Self-Harm: design and development of the supportive response to Self-Harm program. JMIR Formative Res. 2024;8:e50024–e. 10.2196/50024 PMC11185913 38833286

[22] Berger E, Hasking P, Reupert A. We’re working in the dark here’: education needs of teachers and school staff regarding student self-injury. Sch Ment Health. 2014;6(3):201–12.

[23] Heath NL, Toste JR, Sornberger MJ, Wagner C. Teachers’ perceptions of non-suicidal self-injury in the schools. School Ment Health. 2011;3(1):35–43.

[24] Evans R, Parker R, Russell AE, Mathews F, Ford T, Hewitt G, et al. Adolescent self-harm prevention and intervention in secondary schools: a survey of staff in England and Wales. Child Adolesc Ment Health. 2019;24(3):230–8. 31588199 10.1111/camh.12308 PMC6767699

[25] Best R. Deliberate self-harm in adolescence: a challenge for schools. Br J Guidance Couns. 2006;34(2):161–75.

[26] Lee F. Self-harm training in secondary schools: an educational psychology intervention using interpretative phenomenological analysis. Educational Child Psychol. 2016;33(2):105–16.

[27] Timson D, Priest H, Clark-Carter D. Adolescents who self-harm: professional staff knowledge, attitudes and training needs. J Adolesc. 2012;35(5):1307–14. 22705150 10.1016/j.adolescence.2012.05.001

[28] Berger E, Hasking P, Reupert A. Response and training needs of school staff towards student self-injury. Teach Teach Educ. 2014;44:25–34.

[29] Pierret ACS, Anderson JK, Ford TJ, Burn A-M, Review. Education and training interventions, and support tools for school staff to adequately respond to young people who disclose self-harm – a systematic literature review of effectiveness, feasibility and acceptability. 2020. 10.1111/camh.12436 33277965

[30] Burn A-M, Gains H, Anderson JK. A self-harm awareness training module for school staff: co-design and user testing study. JMIR Form Res. 2025;9:e69309. 10.2196/69309 PMC12171642 40455560

[31] Evans R, Hurrell C. The role of schools in children and young people’s self-harm and suicide: systematic review and meta-ethnography of qualitative research. BMC Public Health. 2016;16(1):1–3. 27179765 10.1186/s12889-016-3065-2 PMC4867904

[32] Matthews EL, Townsend ML, Gray AS, Grenyer BFS. Ideal standards for policy on student self-harm: what research and practice tells us. Sch Psychol Int. 2021;42(2):187–209.

[33] Meinhardt I, Cargo T, Te Maro B, Bowden L, Fortune S, Cuthbert S, et al. Development of guidelines for school staff on supporting students who self-harm: a Delphi study. BMC Psychiatry. 2022;22(1):1–14. 36175876 10.1186/s12888-022-04266-7 PMC9520113

[34] Bowden L, Hetrick SE, Cargo T, Woodfield M, Meinhardt I, Clark TC, et al. Maximising the management of self-harm in schools: a collaborative, implementation science approach by secondary schools and child and adolescent mental. Health Serv Mental Health Prev. 2025;37:200391.

[35] Goldberg JM, Sklad M, Elfrink TR, Schreurs KMG, Bohlmeijer ET, Clarke AM. Effectiveness of interventions adopting a whole school approach to enhancing social and emotional development: a meta-analysis. Eur J Psychol Educ. 2019;34:755–82.

[36] Porter BW. W. Understanding the Barriers and Enablers of the Applied Research Collaboration (East of England) Population-in-Focus Approach. A Qualitative Review. 2023.

[37] Barter C, Renold E. The Use of Vignettes in Qualitative Research. 1999. Available from: http://sru.soc.surrey.ac.uk/SRU25.html. Accessed 2019-09-03.

[38] Moore D, Proctor R, Benham-Clarke S, Gains H, Melendez-Torres GJ, Axford N et al. Review of evidence on implementation in education. The Education Endowment Foundation (EEF).; 2024.

[39] Hawton K, Bale L, Brand F, Townsend E, Ness J, Waters K, et al. Mortality in children and adolescents following presentation to hospital after non-fatal self-harm in the multicentre study of self-harm: a prospective observational cohort study. Lancet Child Adolesc Health. 2020;4(2):111–20. 31926769 10.1016/S2352-4642(19)30373-6

[40] Parker R. A small-scale study investigating staff and student perceptions of the barriers to a preventative approach for adolescent self-harm in secondary schools in Wales-a grounded theory model of stigma. Public Health. 2018;159:8–13. 29679862 10.1016/j.puhe.2018.03.016

[41] Venkatesan S. Exploring self-harm: a narrative review of socio-cultural factors. World J Adv Res Reviews. 2024;23:605–15.

[42] Foulkes L, Stapley E. Want to improve school mental health interventions? Ask young people what they actually think. J Philos Educ. 2022;56(1):41–50.

[43] Spratt J, Shucksmith J, Philip K, Watson C. The bad people go and speak to her’: young people’s choice and agency when accessing mental health support in school. Child Soc. 2010;24(6):483–94.

[44] Patte KA, Battista K, Goddard J, Ferrob J, Leatherdale ST. Students’ reasons for being reluctant to seek help for mental health concerns in secondary schools. Cogent Ment Health. 2024;31:1–7. 10.1080/28324765.2023.2298918 PMC12442983 41262653

[45] Dimitropoulos G, Cullen E, Cullen O, Pawluk C, McLuckie A, Patten S, et al. Teachers often see the red flags first: perceptions of school staff regarding their roles in supporting students with mental health concerns. Sch Ment Health. 2022;14(2):402–15.

[46] Meinhardt I, Cuthbert S, Gibson K, Fortune S, Hetrick SE. Young people and adult stakeholders’ reflections on how school staff should support students who self-harm: a qualitative study. J Adolesc. 2022;94(7):969–80. 35880459 10.1002/jad.12078

[47] Fox KR, Bettis AH, Burke TA, Hart EA, Wang SB. Exploring adolescent experiences with disclosing self-injurious thoughts and behaviors across settings. Res Child Adolesc Psychopathol. 2022;50(5):669–81. 34705197 10.1007/s10802-021-00878-x PMC9043038

[48] Dowling S, Doyle L. Responding to self-harm in the school setting: the experience of guidance counsellors and teachers in Ireland. Br J Guidance Counselling. 2017;45(5):583–92.

[49] French ÁGK, Nearchou F, Raftery S, O’Dwyer B, Hennessy E. Parents’ information needs in relation to adolescent self-harm: perspectives of parents and professionals. Arch Suicide Res. 2024;28(4):1131–46. 37950673 10.1080/13811118.2023.2279524

[50] Malterud K, Siersma VD, Guassora AD. Sample size in qualitative interview studies: guided by information power. Qual Health Res. 2016;26(13):1753–60. 26613970 10.1177/1049732315617444

