# Supplementary material for: Principles of practice for a whole school approach to self-harm: a qualitative study
Source: BMC Public Health. 2025 Dec 17;26:267. doi: 10.1186/s12889-025-25538-3 (PMC12822267; doi:10.1186/s12889-025-25538-3)
Supplement: Supplementary file 1 — Supplementary Material 1 [file 12889_2025_25538_MOESM1_ESM.docx]

**Topic guide questions**

**Focus groups with school staff**

**Sorts 1: Your views about self-harm in schools and supporting young people (slide 5)**

- How do you think self harm should be addressed in schools? (taught as part of the curriculum? PSHE, assemblies)
- How comfortable/confident are you having conversations with YP about self-harm? (say, do) who to speak in the school)
- What concerns if any, do you have about talking to a pupil who is self-harming? Do you think some staff will find this difficult to deal with?
- Do you think it’s a good idea to train every member of staff in how to respond? (e.g. admin, teaching and supportive)

**Sorts 2: Sharing thoughts about self-harm in schools and staff’s feelings about supporting young people**

- Have any of you had any training about self-harm?
- How do you think self harm should be addressed in schools?
- How comfortable/confident are you having conversations with YP about self-harm? (say, do) who to speak in the school)
- What concerns if any, do you have about talking to a pupil who is self-harming? Do you think some staff will find this difficult to deal with?
- Do you think it’s a good idea to train every member of staff in how to respond? (e.g. admin, teaching and supportive)
- How can staff be supported?
- What would be helpful for your school in managing self-harm?
- Do you have any guidelines or policies around self-harm?
- Have you come across any prevention programmes?

**Topic guide questions: Focus groups with young people**

Discussion: Experience of Self-Harm in School:

Example scenario presented and YP will be given the chance to respond and feedback.

e.g. A young person asks to speak to their music teacher privately. The young person shares that they have a wound on their arm which is very sore. They inform the teacher that they have harmed themselves.

(1) What do you think the teacher should do/how should they respond? Why do you think so?

(2) What do you think the YP’s attitude/reaction toward teacher’s response? Why do you think so?

- e.g. YP notices a friend has marks on their arm and when they ask their friend about the marks their friend says that they have been self-harming. The YP is very worried about their friend and doesn’t know what to do? They start crying in a lesson and a teacher notices. The teacher asks what is wrong. The YP says that they are worried about their friend and that they think their friend is harming themselves.

(1) What do you think the teacher should do next? Why do you think so?

(2) What do you think the YP’s attitude/reaction toward teacher’s response? Why do you think so?

If a young person wanted to receive some support for their self-harm from their school and not from home, how do you think a school could best support that young person?

- Who, what, when, where?

- A designated person to talk to? Should all staff be approachable and know about self-harm/have the training/skills to support YP?

- A safe space/room to go to?

- Long-term support options/check-ins?

What do you think might be potential barriers that stop young people accessing support for self-harm from their school?

- How do YP speak to friends about SH

- Informing parents?

- Is there a clear procedure for responding to SH?

- Do you think that talking about self-harm in schools (e.g. in lessons or assemblies) will mean that more people will self-harm or start self-harming?

What do school staff need to know about self-harm?

- What do you think that teachers generally think about self-harm?

- What terms should schools/staff use about self-harm?

- Is there anything that staff shouldn’t say when speaking to students about self-harm (as a group or individually)?

- Do particular members of staff need to know more than others (information/training)?

- Do you think school staff need training about how to support young people who self-harm or who are affected by self-harm?

- How should a conversation about self-harm go? How would YP feel best supported?
